# Adipose-Tissue and Intestinal Inflammation – Visceral Obesity and Creeping Fat

**DOI:** 10.3389/fimmu.2014.00462

**Published:** 2014-09-24

**Authors:** Lea I. Kredel, Britta Siegmund

**Affiliations:** ^1^Gastroenterology, Rheumatology, Infectious Diseases, Medical Department I, Charité – Universitätsmedizin Berlin, Berlin, Germany

**Keywords:** adipose-tissue, intestinal inflammation, Crohn’s disease, obesity, adipose-tissue inflammation

## Abstract

Obesity has become one of the main threats to health worldwide and therefore gained increasing clinical and economic significance as well as scientific attention. General adipose-tissue accumulation in obesity is associated with systemically increased pro-inflammatory mediators and humoral and cellular changes within this compartment. These adipose-tissue changes and their systemic consequences led to the concept of obesity as a chronic inflammatory state. A pathognomonic feature of Crohn’s disease (CD) is creeping fat (CF), a locally restricted hyperplasia of the mesenteric fat adjacent to the inflamed segments of the intestine. The precise role of this adipose-tissue and its mediators remains controversial, and ongoing work will have to define whether this compartment is protecting from or contributing to disease activity. This review aims to outline specific cellular changes within the adipose-tissue, occurring in either obesity or CF. Hence the potential impact of adipocytes and resident immune cells from the innate and adaptive immune system will be discussed for both diseases. The second part focuses on the impact of generalized adipose-tissue accumulation in obesity, respectively on the locally restricted form in CD, on intestinal inflammation and on the closely related integrity of the mucosal barrier.

## Introduction

Obesity has become one of the main threats to health worldwide and is outpacing smoking as the primary health hazard (1–4). Due to the increasing clinical and economic significance, fat-tissue has attracted growing scientific attention. Once only recognized as storage for energy, today adipose-tissue is acknowledged as an endocrine organ with multiple functions (5, 6).

The extent of the fat storage is inter individually highly variable and ranges from 5 to 60% of the total body weight. Adipose-tissue is divided into subcutaneous and visceral fat (7).

Several studies reported morphological and functional differences between these adipose-tissue compartments. At least in parts the depot-difference between visceral and subcutaneous fat can be explained by a distinct expression of developmental genes and different adipocyte progenitor cells (8–10). Characteristics of both fat depots are summarized in Table 1.

**Table 1:** **Adipose tissue depots**.

	Subcutaneous adipose tissue (SAT)	Visceral adipose tissue (VAT)
Percentage of the total body fat (7, 142)	∼80%	♂ 10–20% ♀ 5–10%
		The absolute amount of VAT increases with age in both genders
Main depots (7)	- Abdominal	Retro-peritoneal
	- Gluteal	Intra-peritoneal (omental; mesenteric; epiploic)
	- Femoral	
Venous drainage	Dependent on anatomical location	Portal vein
Morphological and functional characteristics (7, 9, 142–151)	- Consists of more preadipocytes per (gram) tissue	- Higher vascularization
	- Higher expression of leptin and CXCL -10	- Greater immune-cell content
		- Adipocytes have an increased metabolic (both lipogenesis and lipolysis) activity
		- Higher expression of pro-inflammatory cytokines (IL-6, IL-8, MCP-1, RANTES, MIP-1α, and PAI-1)
		- Higher adiponectin expression
		- Higher expression of molecules from innate immunity, acute phase response and complement factors, angiotensinogen, and Plasminogen activator inhibitors-1
		- Omentin expression
Metabolic implications (9, 144, 147, 152, 153)		
	- Adipocytes have a higher insulin sensitivity	- Increase is associated with insulin resistance and metabolic syndrome
	- Higher adipogenic ability of the stem cell compartment	- Adipose tissue stem cells over express CD105, Fgf2, and notch target genes
	- Higher intake capacity for free fatty acids and triglycerides	- Surgical removal of VAT in rodents improves insulin sensitivity
	- Major metabolic buffer until a certain “tipping point” → SAT becomes disfunctional due to a positive caloric balance with adipocyte hypertrophy, decreased adipogenesis, and angiogenesis	- Diet and exercise cause preferential fat loss from VAT than SAT

In obesity, a significant expansion of the entire fat-tissue takes place with distinct alterations within the cellular, humoral, and stromal compartment (11–13). The production of pro-inflammatory mediators and the immune-cell infiltration is increased in adipose-tissue of obese compared to lean individuals (5, 6). These adipose-tissue changes and their systemic consequences led to the concept of obesity as a chronic inflammatory state (14). The chronic inflammation results in secondary diseases in the long run and impacts the progression of other illnesses. Especially, visceral adiposity is associated with the development of insulin resistance and correlates strongly with metabolic syndrome (15–17).

**Figure 1:**
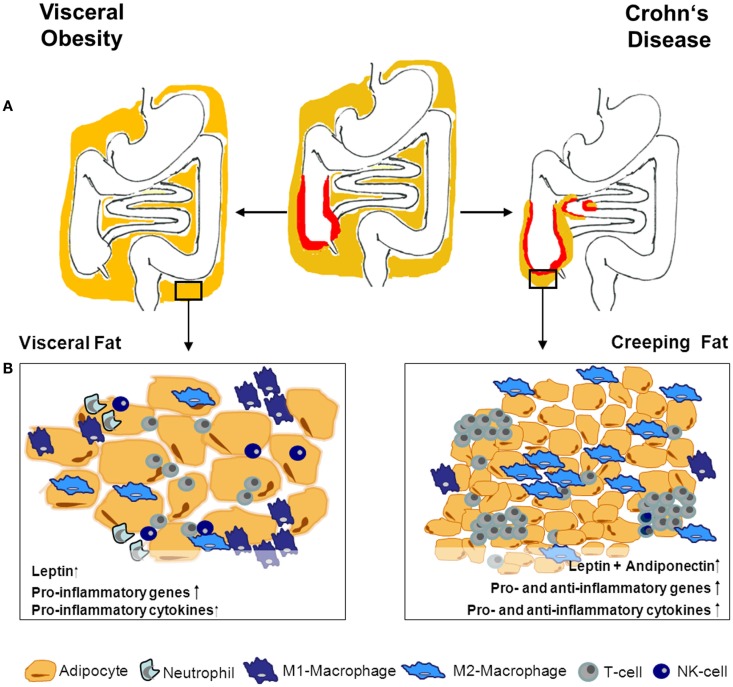
**Adipose tissue morphology and expression profile- differences in obesity and CD**. **(A)** Obesity is defined by a general enlargement of adipose tissue. Especially, the visceral fat-tissue undergoes drastic humoral and cellular changes in obese compared to lean individuals. In Crohn’s disease (CD), adipose tissue accumulation is localized around the inflamed intestinal segments. The interplay between adipose tissue and immune cells during inflammation within the tissue is the subject of ongoing research. Given the differences between adipose tissue in CD and obesity, obese CD patients’ adipose tissue might have varying impact on the intestinal inflammation depending on its localization and reason of development. The different adipose tissue morphology and composition between obesity and creeping fat is illustrated in **(B)**. Adipocytes in obesity are hypertrophic and up-regulate pro-inflammatory mediators. Additionally, the immune-cell compartment within the tissue changes to deprived modulatory/anti-inflammatory cells and increased pro-inflammatory immune cells. Creeping fat is characterized by small hyperplastic adipocytes, with enhanced expression of pro- as well as anti-inflammatory mediators and genes. This is also mirrored in a more balanced increase of different immune cells within the tissue.

Fat accumulation can also be locally restricted. A body weight-independent characteristic hyperplasia of the mesenteric fat-tissue frequently occurs in Crohn’s disease (CD). This so-called creeping fat (CF) enwraps the inflamed segments of the gut and covers more than 50% of the intestinal circumference. Connecting fat accumulation and inflammatory activity, CF correlates with transmural inflammation, fibrosis, muscular hypertrophy, and stricture formation (18–20). Humoral and cellular alterations within the CF are unique and differ from those observed in hypertrophied fat-tissues in obesity (20–22) (Figure 1).

While the existence of CF has been described at the beginning of the last century, the cause of this phenomenon is still unclear.

There is increasing data pointing to a connection between bacterial translocation and the development of CF. Even in the healthy gut bacterial translocation occurs (23, 24), but it is strongly increased in CD (19, 25). Bacteria can trigger adipocytes and preadipocytes proliferation *in vitro* (26). Thus one might speculate, which aggrandized bacterial translocation leads to adipose-tissue hyperplasia in CD (19, 25). Recently, nucleotide-binding oligomerization domain (NOD) variations have been shown to influence adipocyte differentiation. Interestingly, NOD2 variants, which are associated with a higher susceptibility to CD, affect bacterial translocation (27). In CD patients, bacterial mRNA is increased in patients carrying bacterial mRNA compared to controls and the amount of bacterial DNA is related to disease activity. Unfortunately, the author of the study did not give any information regarding the mesenteric fat of these patients. Nevertheless bacteria passing though the intestinal barrier are likely to end up in the mesenteric fat close by, where they might trigger CF development (28).

While the connection between obesity, metabolic, and vascular diseases has been studied intensively, the link between fat accumulation and intestinal inflammation is relatively new. This review aims to examine the association between (intestinal) inflammation and fat accumulation in general and as a local phenomenon. While adipocyte hypertrophy/hyperplasia is accompanied by humoral and cellular changes within the tissue, we particularly discuss the cellular compartments including adipocytes and resident/infiltrating immune cells to define the characteristics of CF versus fat-tissue in obesity.

## Alterations of Fat-Tissue Composition in Obesity and Crohn’s Disease

### Adipocytes

Adipocytes are divided into brown, beige, and white cells presumably covering diverse intermediate forms. White fat cells represent the main type in adipose-tissues of adults (29, 30) therefore this review will focus in this part. Mature white adipocytes contain a large internal fat droplet marginalizing the remaining cytoplasm and nucleus. Adipocytes store the body’s energy supplies, actively produce various mediators and are characterized by their cellular plasticity (31).

Remarkably, the absolute adipocyte number seems to be genetically determined and does not change significantly after the end of the growth phase (32). Tissue enlargement in obesity is primarily due to cellular hypertrophy, rarely to hyperplasia. Enlarged adipocytes in obesity have altered secretory activity with high production of pro-inflammatory cytokines and leptin. Additionally their triglyceride storage is increased (33, 34).

In contrast, CF is a result of adipose-tissue hyperplasia; the adipocytes are significantly smaller and their number is four times increased compared to normal mesenteric fat-tissue (18). While the morphologic changes take place in the adipose-tissue adjacent to the inflamed intestine, the gene expression profile is even altered in visceral fat distant from the inflamed intestinal segment. In obesity especially pro-inflammatory genes are up-regulated, whereas, visceral adipocytes of CD patients show characteristic patterns of increased pro- and anti-inflammatory gene expression (35). In line with this, smaller adipocytes produce less pro-inflammatory mediators (33) and, once activated, adipocytes from the CF are less responsive toward further stimulation (36, 37). Nevertheless, they are highly active producers of different mediators, with significant over-expression of leptin, adiponectin, and resistin as well as of different cytokines and chemokines (22, 38) (Figure 1).

Limiting translation from animal models of intestinal inflammation to human disease, none of the available models show CF-tissue. Still some interesting observation have been made: even though adipose-tissue accumulation does not occur, mononuclear cells infiltrate the mesenteric fat-tissue, adipocyte size decreases, and fibrotic structures appear adjacent to the inflamed murine intestine during acute colitis. Furthermore, mRNA expression of tumor necrosis factor (TNF)α, interleukin (IL)-1β, and IL-6 are up-regulated (39). In trinitrobenzenesulfonic acid-induced colitis in mice and rats TNFα and IL-10 in the mesenteric fat is increased while leptin and adiponectin release is not altered compared to healthy animals (40, 41). Experimental colitis by dinitrobenzenesulfonic acid in Balb/c mice Olivier et al. aimed to establish a model for CF. They successfully induces adipose-tissue accumulation surrounding the ulcerated areas of the inflamed colon (32). This fat accumulation correlated with inflammatory activity and was strictly limited to severe colitis. In contrast to CD patients, animals with less inflammation or regression of their colitis did not show any signs of CF. The hypertrophic mesenteric fat in mice showed high concentrations of IL-6 and monocyte chemotactic protein (MCP)-1 but low adiponectin and leptin levels. Since locally elevated adipokine concentrations are considered hallmarks for the CF, also this model does not completely reflect the distinct characteristics of human CF (42). More recently was shown that in moderately active colitis induced by dinitrobenzenesulfonic acid or dextran sodium sulfate reduced mesenteric fat-tissue was accompanied by increased IL-6 and MCP-1 but decreased adiponectin expression (43).

Besides adipocytes, preadipocytes, stroma cells, and various immune cells are found within the adipose-tissue. Cells of the innate and of the adaptive immune system infiltrate this compartment and their composition changes dependent of the body constitution (44) (Table 2). We again focus on the most relevant cell populations for obesity and CD.

**Table 2:** **Immune-cell characterization in obesity *versus* Crohn’s disease**.

Immune cells	Obesity		Crohn’s disease^a^
	Rodents	Men	Men
Macrophages
- M1	↑ ↑	↑In crown-like structures	↑
- M2	↓	↑ ↑	↑ ↑
Granulocytes
- Eosinophil	↓	n.d.	n.d.
- Neutrophil	↑	n.d.	n.d.
ILC
- NK	n.d.	↑In activated CD56^bright^	n.d.
- ILC2	↓	n.d.	n.d.
T cells	↑	↑	↑
- NKT	↓	↓	n.d.
- CD4^+^ Th1	↑	↑	n.d.
- CD4^+^ Th2	↓	n.d.	n.d.
- CD4^+^ Th17	↓	n.d.	n.d.
- CD8^+^	↑	n.d.	n.d.
- Regulatory FoxP3^+^	↓	↓/↑	n.d.

*^a^No “Rodents” column due to missing immune cells in mesenteric fat-tissue of animal models for Crohn’s disease*.

*n.d., To our best knowledge not done yet*.

### Adipose-tissue macrophages

Macrophages are a heterogeneous cell population, roughly divided into the classically activated, pro-inflammatory M1 and the alternatively activated, immune modulatory M2 subtype, representing two ends of a broad spectrum of plasticity (45, 46). Tissue-resident macrophages are strongly influenced by their environment (47, 48).

Murine adipose-tissue macrophages (ATM) are characterized by high CD14, IL-10, and arginase 1 expression (49). In humans, a broad receptor-expression including the mannose receptor (CD206), various scavenger receptors as well as adhesion molecules like CD163, αvβ5 integrin, CD209, CD200, CD1b, and CD1c has been described for ATM. These macrophages showed high scavenger activity and significant IL-10 and IL-1 receptor antagonist production. In contrast to the “anti-inflammatory” phenotype of some *in vitro*-polarized M2-macrophages, ATM produce large amounts of pro-inflammatory cytokines including TNFα in response to stimulation with either Toll-like receptor ligands or interferon-γ (50, 51). In summary, ATM show an M2 phenotype with pro-inflammatory properties (50, 51).

In obesity and in CD, macrophages accumulate in adipose-tissues. The accumulation within the fat represents an early event in obesity in mice and men (52–54). ATM are a major source of pro-inflammatory mediators in obesity and contribute significantly to the systemic inflammatory status (12, 55–58). ATM can inhibit the effect of insulin in adipocytes leading to systemic insulin resistance via endocrine signaling (54, 59–62). Hence ATM strongly contribute to inflammatory as well as to metabolic consequences of obesity.

Lymphocyte antigen (Ly) 6C^high^ monocytes in the circulation (comparable to CD14^+^ cells in humans) are rapidly recruited to inflammatory sites and sites of tissue remodeling (47). They give rise to monocyte-derived macrophages and dendritic cells. Withal tissue-resident macrophages have to be distinguished from freshly recruited monocytes that differentiate depending on the local milieu (47). Which factors recruit cells into the adipose-tissue and direct polarization of monocytes into their various subsets is not fully understood yet. But ATM accumulation is clearly associated with increased chemokine and adipokine production within the adipose-tissue (63, 64). Especially, MCP-1/CCL2 recruits macrophages into this compartment contributing to insulin resistance (63). Pointing to additional mediators/mechanisms for ATM recruitment, CC-motif receptor (CCR)2-deficiency does not suffice to reduce ATM number and insulin resistance in obese mice (64, 65). CCR5 has also been associated with adipose-tissue inflammation and insulin resistance in mice (66). CXC-motif ligand (CXCL)12, which is increased in diet-induced obesity facilitates ATM recruitment. Blocking the corresponding receptor CXC-motif receptor (CXCR)4 resulted in reduced macrophage accumulation and cytokine release (67). As part of a positive feedback loop between adipose-tissue and bone marrow, adipose-tissue-derived mediators like IL-1β, induce myeloproliferation and monocyte development, contributing to the macrophage accumulation (68). Also hypoxia due to cell hypertrophy takes part in macrophage infiltration in obesity (69, 70). Recently, local proliferation of macrophages within the fat was discovered (71–73). Since only a subset proliferates (74), this does not seem to be the main source of ATM accumulation (73).

It has been known for a while that diet-induced obesity gives rise to a phenotype switch from M2 dominance to that of M1 macrophages in the visceral adipose-tissue of obese mice (49). These macrophages express the M1 markers IL-6, CD11c along with the inducible NO synthase (iNOS) and are typically arranged in crown-like structures around necrotic adipocytes (49, 75, 76).

Human studies regarding ATM alterations in obesity provide contradicting results. On one hand ATM from subcutaneous fat-tissue of obese subjects produce pro-inflammatory markers (57, 77) regressing with weight loss (57, 77, 78). On the other hand M2c-like macrophages with high fibrotic activity and CD150 expression outnumber M1 macrophages forming crown-like structures in the subcutaneous fat of obese patients (79). Correspondingly, ATM from the subcutaneous fat of obese patients were recently found to highly express the anti-inflammatory markers CD163 and IL-10, while TNFα and IL-6 were reduced compared to lean individuals. Since pro-inflammatory markers were equally enhanced, the authors concluded that the adipose-tissue of obese subjects remains more inflamed than in lean subjects (80).

Comparing subcutaneous and visceral fat-tissue, a minor subgroup of ATM localizes within the crown-like structures and the majority appears randomly distributed within the tissues (81). Macrophages in crown-like structures are CD206^+^CD11c^+^ and show features of both subgroups. The other ATM are CD206^+^, CD34^+^ and positive for multiple scavenger receptors but do not express CD11c suggesting their involvement in tissue repair and maintenance. Remarkably, only the amount of CD11c^+^ cells correlated with insulin resistance (81).

A distinct macrophage accumulation is also characteristic for the CF. Although iNOS^+^ M1 macrophages are increased, a far greater accumulation of CD163^+^ and stabilin 1^+^ M2-macrophages suggests a domination of these cells in the CF (82). Specific functions of this cell population are not known so far but *in vitro* data suggest that resident macrophages are strongly influenced by locally increased adipokine levels. Leptin and adiponectin might favor the M2 subtype with high secretory activity for pro- and anti-inflammatory chemokines and cytokines within the CF (82, 83).

### Granulocytes

While macrophages are by far the best characterized immune cells in adipose-tissues, eosinophil granulocytes recently have gained attention. In lean mice, they represent around 5% of the adipose-tissue cells, with decreasing percentages in obesity (84). Eosinophils have been found to substantially modulate adipose-tissue inflammation. Being potent producers of IL-4 and IL-13, they support M2-macrophage polarization and thereby impact glucose homeostasis (84, 85). In obese mice, numbers of eosinophils and M2-macrophages are dramatically reduced within the adipose-tissue. Accordingly, eosinophil-deficient mice have been characterized by high body-fat percentages and metabolic deficiencies like impaired glucose tolerance (84). Eosinophils seem to affect beige progenitors adipocytes and are involved in adaptation of WAT depots to thermogenic challenges (86). However, their recruitment into the fat and their specific function has yet to be elucidated (86). To our knowledge, neither the distribution of eosinophils in adipose-tissues of obese individuals nor in the CF has been studied yet.

Neutrophil granulocytes are rarely found in visceral fat of lean mice but are rapidly recruited into the visceral fat in obesity and seem to take part in impaired insulin sensitivity (87). With neutrophil recruitment as a fast acute response in inflammatory processes in general and the neutrophil chemoattractant IL-8 produced by activated adipocytes and polarized macrophages, recruiting neutrophils is likely to be part of the adipose-tissue inflammation in humans (82, 87). Again, their specific impact in human adipose-tissue inflammation and the generation of CF is not known so far.

### Innate lymphoid cells

Innate lymphoid cells (ILC), cells from the lymphoid line lacking antigen specificity, represent a recently described cell population within the adipose-tissue (88, 89). They are currently classified based on their cytokine expression and specific transcription factors (90). The first group is innate effectors cells with cytotoxic activity comprising natural-killer (NK) and ILC1 cells (91). ILC2, also called natural helper cells, are defined by their production of type-2 cytokines such as IL-4, IL-5, and IL-13 and by the transcription factor GATA3 (92–94). The third group includes ILC3 and lymphoid-tissue inducer (LTi) cells. ILC3 express the NK-cell-activating receptor 46 and RORγt. In contrast to ILC1 cells they do not produce cytotoxic mediators. This subgroup is mainly found in the mucosa of the intestine. ILC3 as well as LTi have been shown to produce IL-17A and IL-22 (91, 94).

Especially, ILC2 cells seem to be critical in adipose-tissue homeostasis (84). By producing IL-5 and IL-13 they recruit eosinophils and M2-macrophages into adipose-tissues (89). Interestingly, IL-25 treatment increased the numbers of ILC2 cells in adipose-tissue of obese mice leading to weight loss and improved glucose tolerance (88). Data on ILC2 in human adipose-tissue are lacking at this point.

A little more is known about NK-cells in fat. NK-cells are categorized according to the intensity of CD56 expression. CD56^bright^ cells are predisposed for IFNγ production and express tissue-homing receptors, whereas, CD56^dim^ cells are present in the peripheral blood and mediate cytotoxicity (95, 96). While numbers of circulating NK-cells are altered in obesity, the number of resident cells in fat-tissues does not change (97). However, the proportion of CD56^bright^ cells within the total NK-cell population seems to be increased in obese subjects and to highly express the activating receptor NKG2D. This activated NK phenotype might indicate their significance for adipose-tissue inflammation that requires further investigation (98).

### T cell compartment

#### Natural-killer T cells

From their innate siblings to the adaptive equivalent: natural-killer T (NKT) cells represent a link between innate and adaptive immunity. Lynch recently provided a detailed overview about their significance in human and murine adipose-tissue (99). NKT cells are divided into type I and type II NKT cells. The well characterized type I NKT cells, also called invariant NKT (iNKT) cells are present in healthy human and murine adipose-tissue. They recognize lipid antigens independent of MHC II via CD1d-restricted presentation by antigen-presenting cells. Upon activation, iNKT cells are fast and potent producers of different chemokines that due to their strong cytotoxic impact activate other immune cells and contribute to anti-tumor activity (99). iNKT cells are primarily tissue-resident cells and comprise T-helper (Th)1-, Th2-, and Th17-like iNKT subsets, whose phenotype and function depends on the local milieu (100, 101). Most adipose-tissue iNKT are CD4^−^, NK1.1^−^, and Th2-polarized cells with regulatory potential and high IL-10 expression (102, 103).

Obesity leads to a decrease of adipose-tissue iNKT in humans and rodents that normally protect from diet-induced obesity and secondary diseases (102–106). By producing regulatory cytokines, enhancing anti-inflammatory macrophages and affecting adipocyte function iNKT cells induce weight loss, and improve fatty liver disease and insulin resistance (105). NKT cell-deficient mice are more susceptible to health risks associated with high-fat diet (HFD) as indicated a fast increase in weight and reduced physical activity (107).

The role of type II NKT cells in adipose-tissue and in obesity-related diseases is less well defined. Even though CD1d^−/−^ mice lacking both NKT populations gain weight and develop metabolic problems, it has been suggested that type II NKT cells are involved in the induction of adipose-tissue inflammation (88, 89). Treatment with IL-25 known to induce ILC2 expansion, resulted in increased infiltration of iNKT and type II NKT cells as well as of other immune cells within the adipose-tissue of obese mice. These cellular changes were accompanied by weight loss and improved glucose tolerance (88). While studies emphasize a protective function for NKT cells regarding obesity and its metabolic consequences, the ultimate proof remains to be provided.

#### T-helper cells

In lean individuals about 10% of the cellular compartment of adipose-tissue consists of CD3^+^ lymphocytes, predominantly of CD4^+^ Th2 or CD4^+^CD25^+^ forkhead box protein 3^+^ regulatory T cells (Treg) (108). However, in obese mice the ratio of CD8^+^ and CD4^+^ T cells is shifted toward CD8^+^ cytotoxic T cells. Within the CD4^+^ lineage Th17 cells as well as Treg are profoundly reduced resulting in a dominance of Th1 cells in this compartment. Supporting this, the absolute cell numbers of Th1 cells are about threefold higher in the visceral fat of diet-induced obese mice as compared to lean controls (108).

In obese humans, the overall number of adipose-tissue T cells is increased (109–111), with a dominance of Th1 over Treg. The ratio correlates with the body mass index (BMI) and changes from 6:1 in lean to 12:1 in obese individuals (112). A positive correlation between the T cell count in adipose-tissue and the BMI of diabetes patients suggests an involvement of these cells in obesity-related metabolic diseases (13, 14).

#### Regulatory T cells

The impact of adipose-tissue Treg has been studied intensively. In lean individuals, Treg accumulate in VF but not in SF (108, 113). Compared to Treg from other organs adipose-tissue Treg have a distinct GATA3^+^, CCR2^+^, KLRG1^+^, and CD103^−^ phenotype with over-expression of CCR1, CCR2, CCR3, CCR5, CCR9, CXCR6, and down-regulation of CCR6, CCR7, CXCR3 (108, 113). Repeated T cell receptor clones are highly suggestive for an adipose-tissue-specific antigen. Besides chemotaxis, e.g., receptor equipment, adipokines, and macrophage-derived mediators seem to contribute to Treg accumulation in the visceral adipose-tissue (108, 113, 114). From a mechanistic point of view, the peroxisome proliferator-activated receptor PPARγ represents a central regulatory pathway for Treg infiltration into the visceral fat (115).

In healthy lean individuals, approximately 15% of the CD4^+^ T cells are Treg; in some inflammatory conditions or malignancies this proportion can increase up to 40% (108, 113, 114). In contrast, as shown in several animal models obesity is accompanied by a pronounced decline of adipose-tissue Treg. The chemokine receptor-expression is altered in adipose-tissue Treg of obese mice with down-regulated CCR1, CCR2, and CXCR6 but increased CXCR3 expression (114). Loss of Treg was associated with higher insulin resistance and risk for diabetes mellitus in these animals (11, 108, 116, 117). Interestingly, aging *per se* seems to reduce the numbers of adipose-tissue Treg paralleled by an increased risk for insulin resistance even in lean mice (114). The anti-diabetic PPARγ agonist pioglitazone increases the Treg numbers in the visceral fat of lean and obese mice (116) underlining that current anti-diabetic treatment directly relies on Treg control (114).

While murine adipose-tissue Treg have been analyzed in detail, little is known about their human counterparts and the results are somewhat conflicting. Three studies found a BMI-dependent decrease of CD4^+^FoxP3^+^ adipose-tissue Treg in the visceral fat of obese humans (11, 108, 112). Zeyda et al. detected higher FoxP3 expression in the same location (118). Treg are involved in gut homeostasis, mucosal integrity, and tolerance (119). While the frequency of Treg in the peripheral blood of patients with inflammatory bowel diseases (IBD) is low, their number is increased within the inflamed mucosa (120, 121). To our knowledge Treg within the adjacent CF have not been quantified yet. With respect to their significance in visceral fat as well as their potential role in IBD further studies regarding this subject are required. Of special interest is that IL-6 can convert Treg into IL-17 producing cells (122) – two cytokines suggested to play a dominant role in the pathogenesis of IBD (123).

To sum up, adipose-tissue hypertrophy is accompanied by profound changes within the resident immune-cell compartment, leading from homeostasis toward a pro-inflammatory local environment. In obesity, these changes have been associated with systemic inflammation and contribute to insulin resistance and type-2 diabetes (49, 75, 87, 102–104, 108, 112). Thus, the inflammatory status of adipose-tissue depends on different factors including location and composition of immune-cell compartments. The last one is strongly influenced by weight gain and obesity resulting in a shift from a homeostatic regulatory environment comprising M2-macrophages, eosinophil granulocytes, NKT cells, and CD4^+^ Treg toward a more pro-inflammatory environment characterized by a dominance of M1 macrophages and a T cell compartment altered toward CD4^+^ Th1 and CD8^+^ cytotoxic subtypes. How these changes are initiated and which individuals are mainly affected remains to be explored.

Having summarized, the current knowledge of the cellular compartment within the adipose-tissue, the last section of this review will serve to outline the interplay of the intestine and the mesenteric fat-tissue.

## Obesity and Barrier Defect

The mucosal barrier consisting of the different cells within the epithelia layer and lamina propria, including (resident) immune cells as well as their cellular products (e.g., mucus, defensins, and cytokines), allows for the absorption of nutritional factors but prevent from increased translocation of bacteria and viruses. Obesity alone seems to suffice to induce an impaired barrier function and intestinal inflammation in rodents (124–126). HFD facilitates translocation of intestinal bacteria and thereby triggers low-grade inflammation in obesity. Emphasizing that the intestinal microbiota forms a prerequisite for the effect of HFD, induced TNFα mRNA and nuclear factor-κB activation was only found in the intestine of specific pathogen free but not of germfree mice (113). In line with these observations, ileal TNFα mRNA expression correlated to weight gain, obesity, and insulin resistance in mice (124). HFD not only affect the microbiota composition in mice with reduced *Lactobacillus* and increased *Oscillibacter* species but equally results in elevated intestinal permeability due to alterations in tight-junction proteins accompanied by a low transepithelial resistance contributing to the barrier defect. In consequence, the mesenteric fat is infiltrated by large numbers of macrophages and associated up-regulation of pro-inflammatory molecules like IL-6 and TNFα (125). These obesity-driven changes are not restricted to dietary-induced obesity. In two genetically driven obese mouse models mucosal barrier defects were concomitant with the increase of bacterial endotoxin and pro-inflammatory cytokines in the portal blood (126).

Taken together these data suggest that the intestinal barrier influenced by genetic risk factors, microbiota, and nutritional factors exert a substantial impact on the development of adipose-tissue inflammation in obesity.

While in obesity mucosal barrier is only mildly affected, CD is characterized by a transmural inflammation with subsequent destruction of the intestinal barrier.

## Obesity and Crohn’s Disease

Even though CD patients rather have normal or subnormal BMI, obesity has become an increasing problem even in this patient collective (127). Whether obesity is a risk factor for CD has not been conclusively answered. A study with 524 obese IBD patients of all ages found a slightly higher risk for developing CD but not for ulcerative colitis. The association was strong for older patients where the influence of environmental factors is expected to be more pronounced in general (128). A European prospective cohort study did not associate obesity to an increased risk of developing CD (129).

Once CD has been established, the course of disease is significantly altered by the patient’s constitution. Overweight CD patients are more prone to active disease and show more frequent anorectal involvement. Consequently, the hospitalization rate is higher and the time until the first surgery is shorter in overweight CD patients compared to lean individuals (128, 130–132). However, there seems to be no significant difference between the overall number of surgical interventions (128, 132, 133).

While children with CD typically present with weight loss and growth retardation, a recent study including nearly 1600 children indicates that approximately 20% of the children with CD were overweight or obese. The rate of obesity in children with ulcerative colitis was 30% and thus comparable to the general population (134). Different from the adult population obesity, had no significant effect on the short-term clinical outcomes of IBD with any perceived difference in disease severity, short-term complications, emergent admissions, or the need for surgical intervention in children. Nevertheless obesity does increase the risk of urinary tract infections and central venous catheter infection in pediatric IBD patients (135).

Besides the progression of disease, also response to therapy seems to be different when comparing lean and obese CD patients. First evidence has been provided by a small single center study that observed a higher risk for loss of response to adalimumab, but not to infliximab treatment of CD in obese patients. The authors hypothesize that the increased body-fat content impairs the efficiency due to changed pharmacokinetic properties. Additionally, high concentrations of circulating pro-inflammatory molecules also altering the therapeutic effect led to the conclusion, which weight-adjusted anti-TNF therapy should be favored in obese patients (136). Using this approach for infliximab treatment did not overcome the shorter time to loss of response in obese IBD patients compared to lean controls, suggesting that weight-adjusted therapy alone is not sufficient (137); a phenomenon also observed in obese spondyloarthritis patients receiving weight-adjusted infliximab therapy (138).

## Diet Versus Obesity – What Drives Inflammation?

As mentioned above, HFD followed by weight gain induces intestinal barrier alterations. Thus not only the events within the fat-tissue and their systemic impact, but also the factors leading to obesity might directly influence inflammatory processes in CD. Especially western (high-fat) diet affects intestinal inflammation in multiple ways from luminal microbiota composition to antigen presentation and change in prostaglandin balance. In a systematic review, Hou et al. outlined the association between pre-diagnosis dietary intake and the risk of developing IBD (139). A higher risk for ulcerative colitis or CD associates with HFD, the increased intake of polyunsaturated fatty acids, omega-6 fatty acids, and meats; and also with saturated fatty acids in CD. While high intake of dietary fiber and fruits decreased the risk of developing CD, ulcerative colitis remained unaffected (139).

Data from animal models support these observations. HFD enhanced intestinal inflammation in a mouse model of experimental colitis (140). Particularly palm-oil based HFD fed to TNF^ΔARE/WT^ mice did not only aggravate intestinal inflammation but promoted expansion of inflammation into the proximal colon, which seems to be of significance given the increased incidence of colonic disease in obese CD patients (127). In this study, disease severity was not associated with development of obesity or metabolic dysfunction, which led to the conclusion that the increased inflammatory activity might not be related to obesity *per se* but is rather induced by the diet itself. Gruber et al. emphasized dietary lipids and so-called metabolic endotoxemia as causes for increased intestinal permeability and pro-inflammatory immune-cell responses (140).

A direct impact on microbiological composition with a rise in the proportion of sulfite-reducing pathobiont bacteria (e.g., *Bilophila wadsworthia*) has been shown for a HFD with mainly saturated fatty acids. These specific bacterial changes were associated with a pro-inflammatory Th1-driven immune response. This diet led to a higher incidence of colitis in genetically susceptible IL-10^−/−^ mice as well as in dextran sulfate sodium -induced colitis. By altering the intraluminal conditions (e.g., host bile acids) saturated fatty acids within the western diet may induce dysbiosis and thereby facilitate an increased inflammatory activity (141).

## Conclusion

Adipose-tissue and its resident cells seem to be highly adaptable and functionally depending on location, cellular composition and dissemination. While obesity is characterized by a widespread increase of adipose-tissue hypertrophy, the fat accumulation in CD is localized and independent of the body weight. Hypertrophic adipocytes have a pro-inflammatory gene expression profile and produce large amounts of pro-inflammatory mediators. Additionally, resident immune cells within the hypertrophic fat-tissue in obesity are primed toward a more pro-inflammatory subtype. These adipose-tissue changes have systemic consequences including elevated serum levels of pro-inflammatory cytokines, C-reactive protein, and leptin. In this way, adipose-tissue inflammation contributes to the development of secondary diseases like diabetes and hypertension in obese individuals.

In contrast secretory adipose-tissue changes in CD seem to be rather focused to the mesenteric fat body. However, a systemic impact can be equally suggested, since C-reactive protein that is found increased systemically in active disease, can be produced by the mesenteric fat-tissue. Interestingly, adipokine levels are only locally elevated. Yet, the gene expression profile as well as mediator secretion of small adipocytes within the CF is characterized by both anti- as well as pro-inflammatory capacities, pointing to a more balanced situation within the fat-tissue. Thus adipose-tissue inflammation in the CF might directly modulate, possible even control, intestinal inflammation.

While there are convincing mouse data to adipose-tissue changes in obesity, human data are still rare. Especially in regard to the differences between mouse and man (e.g., macrophage distribution in adipose-tissue) there is a need for comparative studies. Additional studies are required to characterize resident immune cells within the fat in inflammatory conditions especially in the visceral and CF. While knowledge about alterations in obese individuals might help to reveal novel strategies for the fight against obesity, a better understanding of the role of the adipose-tissue might also help to define novel therapeutic strategies for CD.

## Conflict of Interest Statement

The authors declare that the research was conducted in the absence of any commercial or financial relationships that could be construed as a potential conflict of interest.
